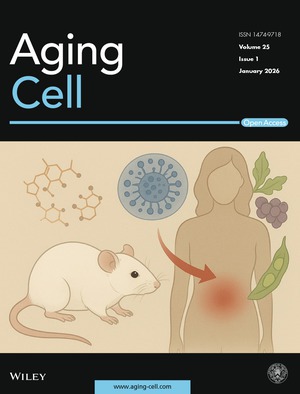# Featured Cover

**DOI:** 10.1111/acel.70414

**Published:** 2026-02-15

**Authors:** Nisansala Chandimali, Jaehoon Bae, Sun Hee Cheong, Seon Gyeong Bak, Yeon‐Yong Kim, Ji‐Su Kim, Seung‐Jae Lee

## Abstract

Cover legend: The cover image is based on the article *Humanized Mouse Models as a Cellular Platform for Investigating Immune‐Hormonal Crosstalk and Therapeutic Strategies in Menopause* by Nisansala Chandimali *et al.,* https://doi.org/10.1111/acel.70313.